# Patients’ preferences for follow-up after endometrial cancer surgery

**DOI:** 10.2340/1651-226X.2026.45981

**Published:** 2026-06-27

**Authors:** Linn Renman, Lotta Andréen, Sahruh Turkmen

**Affiliations:** a Department of Obstetrics and Gynecology, Sundsvall County Hospital, Sundsvall, Sweden; b Department of Clinical Sciences, Obstetrics and Gynecology, Sundsvall Research Unit, Umeå University, Umeå, Sweden

**Keywords:** Women, endometrial neoplasm, quality of life, patient-centered care, follow-up studies

## Abstract

**Background and purpose:**

To compare follow-up preferences and health-related quality of life between newly operated endometrial cancer patients and those already enrolled in a traditional hospital-based follow-up program, and to explore whether preferences differ depending on follow-up experience.

**Patient/material and methods:**

In this prospective cross-sectional study, patients who underwent primary surgery for endometrial cancer in northern Sweden were included. Group 1 consisted of newly operated patients recruited before entering follow-up, and Group 2 included patients already participating in the standard hospital-based follow-up program. Participants completed questionnaires on sociodemographic characteristics, follow-up preferences, and quality of life (EORTC QLQ-C30). Clinical data were obtained from medical records. The primary outcome was follow-up preference; secondary outcomes included quality of life and associations with clinical and demographic variables.

**Results:**

Of the 121 invited patients, 92 (76%) responded, and 90 were included in the analysis (48 in Group 1; 42 in Group 2). The groups were comparable in baseline characteristics. Newly operated patients demonstrated more diverse follow-up preferences, with 51.1% preferring physician-led hospital visits compared to 82.9% in Group 2 (*p = 0.035*). Global quality of life did not differ significantly (*p* = 0.165), although Group 1 reported lower functional scores, particularly in role and emotional functioning. Appetite loss was the only symptom that differed significantly between groups.

**Interpretation:**

Newly operated patients are more open to alternative follow-up models despite similar overall quality of life. Integrating patient preferences into follow-up care may enhance personalization and sustainability.

## Introduction

Endometrial cancer is a malignant tumor that originates from the endometrial lining of the uterine corpus. It is the most common gynecological cancer in high-income countries, with a rising incidence due to lifestyle factors such as obesity and diabetes [1–5]. Endometrial cancer is generally associated with a favorable prognosis and low mortality [1–5]. It is often diagnosed at an early stage; in Sweden, approximately 66% of cases are detected at stage I [1]. The risk of recurrence is low in early-stage disease (3%), and the majority (70%) of recurrences are symptomatic [1, 6, 7].

Providing post-treatment follow-up services for an increasing number of cancer survivors places additional pressure on healthcare systems. According to Swedish healthcare guidelines for endometrial cancer, hospital-based clinical follow-up by a gynecologist or gynecological oncologist is recommended for 5 years after treatment, with at least two visits per year during the first 2 years, followed by one visit per year during the subsequent 3 years [1]. The purpose of hospital-based follow-up is to detect recurrence at an early stage and to monitor patients’ rehabilitation needs [1, 8]. However, previous studies have not demonstrated a survival benefit for patients attending scheduled hospital follow-up visits after endometrial cancer treatment, and early detection of recurrence does not appear to improve outcomes [3, 6, 9–14]. Furthermore, there is no clear evidence regarding the optimal frequency of follow-up visits, and follow-up protocols vary widely internationally [15].

As much as 70% of all tumor-related expenditures are allocated to follow-up [16]. Several recent European studies have explored alternative follow-up strategies for patients with low-risk endometrial cancer, including telephone-based follow-up (TFU) [17, 18], reduced follow-up programs [19], and patient-initiated follow-up (PIFU) [20–23]. These studies have shown that non-traditional follow-up strategies can reduce costs for both healthcare services and patients, while maintaining patient satisfaction and quality of life [24].

An earlier British study by Beaver et al. [25] investigated low-risk endometrial cancer patients’ preferences for hospital-based versus TFU. The results indicated that patients tended to prefer the follow-up model with which they were familiar. However, no study has examined endometrial cancer patients’ preferences for post-treatment follow-up prior to the initiation of a follow-up program.

We additionally explored quality of life and International Federation of Gynecology and Obstetrics (FIGO) stage as potential determinants of follow-up preferences. Patients experiencing greater symptom burden, impaired functioning, or more advanced disease may be expected to prefer more intensive physician-led follow-up, whereas patients with fewer symptoms may be more receptive to alternative models of care.

This study aimed to investigate patients’ preferences for follow‑up plans after surgical treatment for endometrial cancer prior to the initiation of follow‑up, and to assess their quality of life in relation to cancer stage and follow‑up preference. These preferences and quality‑of‑life measures were compared with those of a similar group of patients already receiving hospital‑based follow‑up. We hypothesized that women with endometrial cancer would prefer hospital‑based follow‑up appointments with a gynecologist over alternative follow‑up methods.

## Patients/material and methods

### Study design and population

We conducted a prospective cross-sectional study of patients with endometrial cancer who underwent primary surgical treatment. This study was approved by the Swedish Ethical Review Authority (Dnr 2024-01635-01).

Patients from northern Sweden who had undergone primary surgical treatment for endometrial cancer were included after receiving written information about the study and providing written informed consent. Patients were divided into two groups. Group 1 comprised patients in the planning phase, prior to entering the follow-up program, and were recruited immediately after primary surgery at Norrland University Hospital, Umeå, Sweden, over a 10-month period beginning in September 2024. Group 2 included patients already enrolled in the traditional Swedish hospital-based follow-up program and consisted of patients receiving hospital-based follow-up at Sundsvall County Hospital, Sweden. Both groups were drawn from the same regional catchment area in northern Sweden but were recruited at different hospitals. Group 1 patients were treated and recruited at the tertiary referral center (Norrland University Hospital), whereas Group 2 patients received routine follow-up at Sundsvall County Hospital. Thus, Group 1 represents a single-center cohort prior to follow-up initiation, while Group 2 reflects standard follow-up care at a separate county hospital within the same healthcare region.

Inclusion criteria comprised patients who met the following conditions: a diagnosis of endometrial cancer, undergoing primary surgical treatment for endometrial cancer, residence in northern Sweden, ability to provide written informed consent after receiving information about the study, ability to complete written questionnaires in Swedish, and status as either in the planning phase before entering follow-up program (Group 1) or already enrolled in the traditional hospital-based follow-up program (Group 2).

Exclusion criteria included the following: inability to complete written questionnaires due to visual impairment or insufficient proficiency in the Swedish language; receipt of chemotherapy prior to surgery; and for Group 2, a history of cancer recurrence.

### Data collection

Data were collected using two complementary methods, patient questionnaires and chart review, to capture both patient-reported and clinical information.

Patient questionnaires, accompanied by information about the study and a written consent form, were sent by post along with a pre-paid return envelope to facilitate response and maximize participation. The questionnaires collected sociodemographic information, including educational level, marital status, and employment status, as well as age and street address to calculate the distance to hospital. This information enabled exploration of potential associations between demographic factors, geographic accessibility, and patient preferences or quality of life outcomes. The questionnaire package also included a question on preference on follow-up adapted from Beaver et al. [25], in which patients were asked to rank their top three preferences for follow-up from a list of 10 options (Appendix 1). Collecting these data allowed assessment of patient-centered priorities in follow-up care. Additionally, the questionnaires incorporated the European Organisation for Research and Treatment of Cancer Quality of Life Questionnaire Core 30 (EORTC QLQ-C30 version 3.0) [26]. The QLQ-C30 includes a global health status scale, five functional scales (physical, role, emotional, cognitive, and social), three symptom scales (fatigue, nausea/vomiting, and pain), and six single items regarding symptoms (dyspnea, insomnia, appetite loss, constipation, diarrhea, and financial difficulties).

Chart review involved systematically extracting clinical information from medical records, including primary surgery, FIGO stage [27], histological type, adjuvant treatment (if any), comorbidities, body mass index (BMI), and American Society of Anesthesiologists (ASA) classification [28]. This method provided objective clinical data and baseline patient characteristics relevant for analyzing outcomes and treatment patterns. All data extraction was conducted using a standardized form to ensure consistency and minimize errors.

### Study outcomes

The study outcomes were organized by group. The primary outcome was patient preferences for follow-up after surgical treatment for endometrial cancer. Preferences in Group 1 were assessed before the start of follow-up, whereas preferences in Group 2 were assessed during ongoing hospital-based follow-up.

Secondary outcomes included quality of life, measured using the EORTC QLQ-C30; the association between quality of life and cancer stage (FIGO stage); and the association between quality of life and follow-up preference, adjusted for clinical and/or demographic confounders, including age, comorbidities, BMI, educational level, marital status, and employment status.

Exploratory outcomes included the comparison of quality of life and follow-up preferences between the two groups, as well as the proportion of patients preferring hospital-based follow-up versus alternative methods.

### Statistical analysis

All analyses were performed using the Statistical Package for the Social Sciences (SPSS) software, version 29 (IBM, USA). Descriptive statistics were used to summarize participant characteristics. Normality of data distributions was assessed using the Shapiro-Wilk test. Continuous data are presented as median and interquartile range, and categorical data as percentages.

The Mann-Whitney U test was used to compare continuous variables between groups, and the Pearson’s chi-square test was applied for categorical variables. For all analyses, the significance level was set at 5% (*p* < 0.05).

Results from the EORTC QLQ-C30 were scored according to the EORTC QLQ-C30 scoring manual [29], generating scores ranging from 0 to 100 for functioning, global health status, and symptoms. Higher scores on functional scales and the global health status scale indicate better functioning and quality of life, whereas higher scores on the symptom scales/items indicate a higher level of symptoms.

To account for potential confounders, regression analyses were performed. Covariates were selected a priori based on clinical relevance and previous literature indicating potential associations with healthcare preferences, quality of life, or disease severity. Linear regression was used for continuous outcomes, such as quality-of-life scores, and logistic regression was applied for categorical outcomes, such as follow-up preference. The multivariate logistic regression analysis included adjustment for relevant covariates, including age, BMI, education level, employment status, marital status, comorbidities, histological type, FIGO stage, adjuvant treatment, surgery type, postoperative complication, ASA classification (American Society of Anesthesiologists Physical Status Classification System), and QLQ subgroups. When the independent variable was nominal or ordinal, we applied dummy coding to convert the categories into binary indicators, allowing their inclusion in the multivariable regression model. Given the exploratory nature of the quality-of-life analyses and the large number of comparisons performed across multiple EORTC QLQ-C30 domains, findings from individual statistically significant comparisons should be interpreted with caution because of the increased risk of type I error.

#### Sample size

Based on a previous study by Beaver et al. comparing preferences for hospital- versus TFU, 81.04% of participants preferred hospital follow-up, while 23.69% preferred telephone follow-up [25]. Using a power of 0.9 and a significance level (alpha) of 0.05, the estimated sample size required was 28 patients, with 14 patients in each group.

## Results

**Figure 1:**
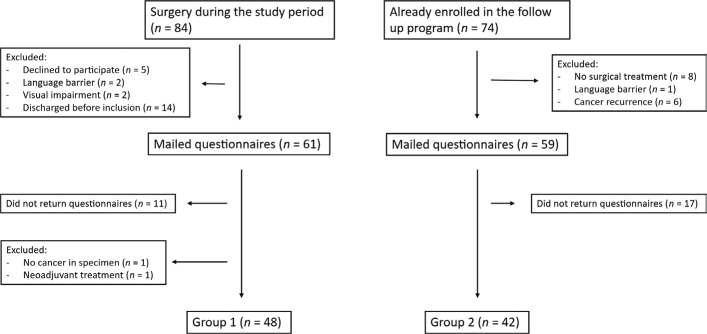
Flowchart of recruitment to the study.

A total of 158 patients were assessed for eligibility, and after applying the inclusion and exclusion criteria, 120 women were invited to participate and received the questionnaire package. Of these, 92 (77%) returned the questionnaires, including 50 patients in the newly operated group (Group 1) and 42 in the control group (Group 2). Two patients in Group 1 were subsequently excluded – one because of no cancer was found in the surgical specimen and one who had received neoadjuvant chemotherapy – resulting in a final study population of 90 patients: 48 in Group 1 and 42 in Group 2 (Figure 1).

Participant characteristics are summarized in Table 1. Histopathologic classification and FIGO stage distribution were comparable between the groups; endometrial histology and early-stage disease predominated in both groups, with no statistically significant differences observed. No significant differences were found between the groups in demographic, social, or cancer-related characteristics.

**Table 1:** Participant characteristics.

Characteristic	Group 1 (*n* = 48) Median (IQR)/*n* (%)	Group 2 (*n* = 42) Median (IQR)/*n* (%)	*p*
Age	68 (61–75)	72 (61.75–78.25)	0.202^1^
BMI	31 (26.25–36)	33 (29–40)	0.196^1^
Distance to hospital (km)	20 (6–57.5)	19 (8.8–49)	0.487^1^
Days to response after surgery	16,5 (12–21.5)	996 (528–1495)	< 0.001^1^
Married/Cohabitant Yes No	33 (71.7%) 13 (28.3%)	29 (70.7%) 12 (29.3%)	0.917^2^
Employed Yes No	16 (13.8%) 30 (65.2%)	9 (22%) 32 (78%)	0.187^2^
Education Basic/High school Higher education	33 (71.7%) 13 (28.3%)	26 (65%) 14 (35%)	0.502^2^
Comorbidity No Cardiovascular disease Other	11 (22.9%) 26 (54.2%) 11 (22.9%)	8 (19%) 26 (61.9%) 8 (19%)	0.760^2^
ASA-class at surgery 1 2 3 4	8 (16.7%) 30 (62.5%) 8 (16.7%) 2 (4.2%)	9 (21.4%) 24 (57.1%) 8 (19%) 1 (2.4%)	0.733^1^
Type of surgery Laparoscopic Laparotomy Vaginal	43 (89.6%) 5 (10.4%) 0	35 (83.3%) 6 (14.3%) 1 (2.4%)	0.468^2^
Complication at surgery Yes No	1 (2.1%) 47 (97.9%)	1 (2.4%) 41 (97.6%)	0.924^2^
Histopathologic classification Endometrial Other	38 (79.2%) 10 (20.8%)	37 (88.1%) 5 (11.9%)	0.257^2^
FIGO stage 1 2 3 4	36 (75%) 2 (4.2%) 6 (12.5%) 4 (8.3%)	37 (88.1%) 2 (4.8%) 3 (7.1%) 0	0.086^1^
Adjuvant treatment Yes No	14 (29.2%) 34 (70.8%)	7 (16.7%) 35 (83.3%)	0.162^2^
QLQ Function Symptom Global	76 (61–87) 17 (9–37) 67 (50–81)	87 (69–96.5) 15 (5.5–30.5) 67 (50–83)	0.012^1^ 0.424^1^ 0.165^1^

BMI: body mass index; ASA: American Society of Anesthesiologists; FIGO 2009: International Federation of Gynecology and Obstetrics; QLQ: European Organisation of Research and Treatment of Cancer Quality of Life Questionnaire Core 30. Group 1: newly operated patients; Group 2: patients in ongoing hospital-based follow-up; *n*: number.

1 Mann-Whitney,

2 Chi-square.

Patients’ follow-up preferences showed that, as the first-choice preference, 51.1% of patients in Group 1 preferred hospital appointments with a doctor, compared with 82.9% in Group 2 (*p* = 0.035). Patients in Group 1 demonstrated greater diversity in follow-up preferences than those enrolled in the traditional hospital-based program. No differences were observed between the groups for second- and third-choice preferences; however, higher proportions of missing data were noted for these preferences, with 22% missing responses for second-choice preferences and 24% for third-choice preferences, compared with first-choice preferences (Table 2).

**Table 2:** Distribution of patients’ top three follow-up preferences by study group.

Preference option *	Group 1 *n* (%)										Group 2 *n* (%)										*p*-value
	1	2	3	4	5	6	7	8	9	10	1	2	3	4	5	6	7	8	9	10	
Preference number 1 *n* = 88	24 (51.1)	2 (4.3)	5 (10.6)	0	5 (10.6)	5 (10.6)	1 (2.1)	2 (4.3)	3 (6.4)	0	34 (82.9)	0	1 (2.4)	0	0	3 (7.3)	0	2 (4.9)	0	1 (2.4)	0.035
Preference number 2 *n* = 69	5 (11.4)	7 (15.9)	5 (11.4)	2 (4.5)	11 (25)	11 (25)	1 (2.3)	2 (4.5)	0	0	1 (4.0)	6 (24)	3 (12)	1 (4)	4 (16)	10 (40)	0	0	0	0	0.655
Preference number 3 *n* = 67	3 (7.1)	9 (21.4)	5 (11.9)	4 (9.5)	11 (26.2)	6 (14.3)	2 (4.8)	2 (4.8)	0	0	2 (8)	9 (36)	3 (12)	1 (4)	7 (28)	2 (8)	0	0	0	1 (4)	0.592

Group 1: newly operated patients; Group 2: patients in ongoing hospital-based follow-up; *n*: number.

* • Preference 1: Hospital doctor • Preference 2: Hospital specialist nurse • Preference 3: Telephone doctor • Preference 4: Telephone specialist nurse • Preference 5: Initial hospital doctor visit, then telephone specialist nurse follow-up • Preference 6: Initial hospital doctor visit, then hospital specialist nurse follow-up • Preference 7: General Practitioner follow-up • Preference 8: Discharge with patient-initiated contact if needed • Preference 9: Other • Preference 10: No preference

*P*‑value indicates differences between groups.

Quality of life was assessed using the EORTC QLQ-C30 (Table 3). No significant differences were observed between the groups in global quality of life (*p* = 0.165) or overall symptom scores. Overall functional scores were significantly lower in Group 1 than in Group 2 (76 vs. 87; *p* = 0.012). In the functional subscales, role functioning (*p* < 0.001) and emotional functioning (*p* = 0.042) were significantly lower in Group 1, whereas physical, cognitive, and social functioning did not differ. Among the symptom subscales, appetite loss was the only symptom with a significant difference, with higher scores in Group 1 (*p* = 0.047); all other symptoms, including fatigue, nausea, pain, dyspnea, insomnia, constipation, diarrhea, and financial difficulties, were similar between the groups.

**Table 3:** Comparison of health-related quality of life scores between study groups, measured using the European Organisation for Research and Treatment of Cancer Quality of Life Questionnaire Core 30 (EORTC QLQ-C30).

QLQ-C30	Group 1 (*n* = 48) Median (IQR)	Group 2 (*n* = 42) Median (IQR)	*p* *
Global quality of life (0–100)	67 (50–81)	67 (50–83)	0.165
Function (overall) Physical functioning (0–100) Role functioning (0–100) Emotional functioning (0–100) Cognitive functioning (0–100) Social functioning (0–100)	76 (61–87) 80 (60–93) 67 (17–83) 75 (58–92) 100 (83–100) 83 (50–100)	87 (69–96.5) 80 (63.5–96.5) 83.3 (67–100) 83 (67–100) 100 (83–100) 100 (67–100)	0.012 0.443 < 0.001 0.042 0.993 0.062
Symptoms (overall) Fatigue (0–100) Nausea and vomiting (0–100) Pain (0–100) Dyspnea (0–100) Insomnia (0–100) Appetite loss (0–100) Constipation (0–100) Diarrhea (0–100) Financial difficulties (0–100)	17 (9–37) 44 (11–56) 0 (0–17) 33 (0–50) 0 (0–33) 33 (0–33) 0 (0–33) 0 (0–33) 0 (0–33) 0 (0–0)	15 (5.5–30.5) 33 (11–56) 0 (0–0) 17 (0–50) 33 (0–50) 33 (0–33) 0 (0–0) 0 (0–33) 0 (0–16.5) 0 (0–0)	0.424 0.285 0.599 0.438 0.308 0.673 0.047 0.171 0.190 0.990

Group 1: newly operated patients; Group 2: patients in ongoing hospital-based follow-up. *n* = number.

* Mann-Whitney U-test.

When quality of life was analyzed according to the FIGO stage, no significant differences were observed between patients with early-stage and more advanced-stage endometrial cancer, with global, functional, and symptom scores remaining comparable (Supplementary Table 2).

To assess the influence of potential confounding factors on patients’ first-choice follow-up preference, a multivariate logistic regression analysis was performed in both groups. In Group 1, BMI (odds ratio [OR] = 1.65, 95% confidence interval [CI]: 1.011–2.699, *p* = 0.045), employment status (OR = 0.00, 95% CI: 0.000–0.709, *p* = 0.045), and QLQ-symptom score (OR = 0.81, 95% CI: 0.675–0.987, *p* = 0.036) were significantly associated with choosing hospital visits with a doctor as the first-choice follow-up. However, the estimate for employment status was considered unstable due to possible complete or quasi-complete separation; therefore, the OR = 0.00 (95% CI: 0.000–0.709) should be interpreted with caution. In Group 2, no factors were significantly associated with this preference.

Correlations between continuous parameters were also examined. In Group 1 (newly operated patients), age was significantly correlated with QLQ-Function (*r* = 0.321, *p* = 0.028), whereas no such correlation was observed in Group 2. As expected, significant correlations were observed among QLQ-Function, QLQ-Symptom, and QLQ-Global scores in both groups: in Group 1, QLQ-Function and QLQ-Symptom (*r* = −0.729, *p* ≤ 0.001), QLQ-Function and QLQ-Global (*r* = 0.679, *p* ≤ 0.001), and QLQ-Symptom and QLQ-Global (*r* = −0.711, *p* ≤ 0.001); in Group 2, QLQ-Function and QLQ-Symptom (*r* = −0.773, *p* ≤ 0.001), QLQ-Function and QLQ-Global (*r* = 0.792, *p* ≤ 0.001), and QLQ-Symptom and QLQ-Global (*r* = −0.639, *p* ≤ 0.001).

No associations were observed between quality of life and patients’ follow-up preferences in either group. The only significant association was in the previously operated group, where increasing time since surgery was associated with a greater preference for hospital-based follow-up options (B = 0.002, 95% CI: 0.000–0.004).

## Discussion and conclusion

This study investigated endometrial cancer patients’ preferences for follow-up after treatment, including both newly operated patients and those already enrolled in a traditional hospital-based follow-up program. Our findings indicate that newly operated patients are more open to diverse follow-up methods compared with patients who have already begun a structured program. Only about half of the newly operated patients (51.1%) selected hospital-based appointments with a physician as their first choice, compared with 82.9% of patients in the traditional program. This difference suggests that patients who have not yet been exposed to a specific follow-up structure are more receptive to alternative models of care, creating an opportunity to redesign follow-up pathways in ways that align with patient expectations while maintaining clinical safety.

To our knowledge, this is the first study to examine follow-up preferences in newly operated endometrial cancer patients prior to their entry into a follow-up program. Consistent with our hypothesis, these patients demonstrated greater openness to alternative follow-up models than those already enrolled in the traditional program, who preferred to continue with physician-led hospital visits. Previous studies evaluating TFU and PIFU have reported high acceptability and patient satisfaction [23, 25, 30–32]. Combined with the well-established evidence that routine physical follow-up visits do not improve outcomes or survival in endometrial cancer [6, 9–11, 14], our findings support the feasibility of implementing alternative follow-up strategies. The clear difference in preference between newly operated patients and those already in structured follow-up underscores the influence of prior experience on patient expectations and highlights the value of offering flexible models early in the survivorship trajectory.

The follow-up schedule recommended by the Swedish national guidelines requiring at least two visits per year to a gynecologist or gynecologic oncologist during the first 2 years, followed by annual visits for 3 additional years [1] places substantial demands on an already strained healthcare system, especially given the growing number of cancer survivors. Our results suggest that individualized follow‑up, based on recurrence risk and patient preference, may offer a more sustainable and resource‑efficient alternative. Low‑risk patients open to TFU, PIFU, or reduced‑frequency hospital visits could safely be offered these options, while those with higher risk or greater need for reassurance may continue with physician‑led appointments. Integrating patient preferences with clinical risk stratification would enable more personalized follow‑up type and interval, potentially improve patient satisfaction, and reduce unnecessary hospital visits.

Patients who had already experienced a particular follow‑up model tended to prefer continuing with the approach they were familiar with. This finding aligns with previous research, showing that familiarity strongly influences patient preference across follow‑up regimens [18, 25, 31]. Such stability suggests that once patients become accustomed to a structure, they may perceive it as safer or more reassuring, even when alternatives are equally effective.

However, the observed differences in follow-up preferences should be interpreted with caution. Although familiarity with a specific follow-up model is likely to have influenced patients’ choices, other factors may also have contributed. These may include individual perceptions of safety and reassurance, previous experiences with healthcare, personal coping strategies, expectations regarding recurrence detection, and preferences for convenience or autonomy in care. Furthermore, the groups differed substantially in time since surgery, which may have influenced patients’ perspectives on follow-up needs. Consequently, the present study cannot determine the relative importance of familiarity compared with other factors influencing follow-up preferences.

Another key finding was the overall similarity in global quality of life between the two groups, as measured by the EORTC QLQ-C30. No significant differences were observed in global health status, and quality of life was not associated with follow-up preference in either group. These findings suggest that overall perceived quality of life does not appear to be a major determinant of patients’ preferences for follow-up strategy after endometrial cancer surgery. Some differences were observed in specific functional domains, with lower role and emotional functioning in the newly operated group. However, these differences are likely explained by the very short interval between surgery and questionnaire completion in Group 1 and are consistent with expected short-term postoperative recovery.

Despite lower emotional functioning in the newly operated group, this did not translate into a stronger preference for physician-led follow-up, suggesting that short-term emotional well-being is not a key determinant of follow-up preferences. No differences were observed in global quality of life between groups, and quality-of-life measures did not explain variation in follow-up preferences. Overall, these findings indicate that quality of life has limited influence on patients’ follow-up preferences in this cohort.

Interestingly, despite lower emotional functioning shortly after surgery, newly operated patients did not demonstrate a stronger preference for physician-led follow-up. This suggests that emotional distress alone may not determine follow-up preferences, and that other factors, such as convenience, autonomy, previous healthcare experiences, or expectations regarding recurrence detection, may be equally important.

Previous studies on quality of life after the endometrial cancer treatment have shown that global quality of life remains stable during long‑term follow‑up [33–35]. Research on robotic‑assisted laparoscopic hysterectomy, the procedure most Group 1 patients underwent, has demonstrated short‑term declines in global quality of life during the first 1–2 postoperative weeks, with recovery to baseline within 5 weeks to 3 months [34, 35]. In our study, we did not observe lower global quality of life in the newly operated group compared with the control group, although the cross‑sectional design limits conclusions about long‑term recovery. It remains possible that global quality of life in Group 1 would continue to improve over time, as suggested by previous longitudinal studies.

A major strength of this study is that follow-up preferences were assessed before patients entered a follow-up program, thereby capturing preferences prior to exposure to a specific surveillance model. This design reduces the risk of experience-driven bias and provides insight into patients’ initial expectations regarding follow-up after endometrial cancer treatment. Inclusion of patients across all FIGO stages further enhances the clinical relevance of the findings.

Several limitations should be considered. First, the cross-sectional design limits the ability to assess changes in preferences over time as patients gain experience with different follow-up strategies. This may influence interpretation of whether early preferences are stable or transient; however, the impact on the primary outcome is considered moderate. Second, all patients in Group 1 were recruited from a single tertiary referral center, which may limit external validity. While this may introduce some selection bias, the study population reflects routine clinical care within the regional healthcare system, suggesting a moderate impact on generalizability. Third, patients who were unable to complete Swedish-language questionnaires were excluded. This may limit generalizability, as individuals with different linguistic or cultural backgrounds may have different healthcare expectations, levels of trust in the healthcare system, and preferences regarding follow-up structure. The potential impact on results is considered moderate to high with respect to external validity, but unlikely to affect the internal comparisons between study groups. Finally, missing data for second- and third-choice preferences limited interpretation of the full hierarchy of patient preferences, although the primary outcome (first-choice preference) was well captured.

Newly operated patients with endometrial cancer demonstrate a clear openness to a variety of follow‑up models, in contrast to patients already enrolled in traditional hospital‑based follow‑up. This is noteworthy despite the absence of differences in global QLQ‑C30 scores between the two groups, indicating that openness to alternative follow‑up is not driven by disparities in overall quality of life. These findings suggest that patient preferences should be actively considered when revising national follow‑up recommenda tions. Incorporating patient‑centered options particularly for those early in the postoperative period may support the development of a more flexible, individualized, and sustainable follow‑up program.

## Supplementary Material





## Data Availability

The data generated during the current study are available from the corresponding author upon reasonable request.
