# Supplementary material for: Patients’ preferences for follow-up after endometrial cancer surgery
Source: Acta Oncol. 2026 Jun 27;65:45981. doi: 10.2340/1651-226X.2026.45981 (PMC13421151; doi:10.2340/1651-226X.2026.45981)
Supplement: Supplementary file 1 [file AO-65-45981-s1.pdf]

Supplementary material has been published as submitted. It has not been copyedited, or typeset by Acta Oncologica

Appendix 1. Patients were asked to rank their top three follow-up preferences from a list of ten predefined surveillance options (Rank 1–3).

I would prefer appointments at the hospital with a doctor
I would prefer appointments at the hospital with a specialist nurse
I would prefer telephone appointments with a doctor
I would prefer telephone appointments with a specialist nurse
I would prefer my first hospital appointment with a doctor and then telephone appointments with a specialist nurse
I would prefer my first hospital appointment with a doctor and then hospital appointments with a specialist nurse
I would prefer to have my follow-up appointments with my General Practitioner (GP)
I would prefer to be discharged from follow-up after treatment as long as I know who to contact if I have problem
Other (please explain)
No preferences
