# Supplementary material for: Patients’ preferences for follow-up after endometrial cancer surgery
Source: Acta Oncol. 2026 Jun 27;65:45981. doi: 10.2340/1651-226X.2026.45981 (PMC13421151; doi:10.2340/1651-226X.2026.45981)
Supplement: Supplementary file 2 [file AO-65-45981-s2.pdf]

Supplementary material has been published as submitted. It has not been copyedited, or typeset by Acta Oncologica

Supplementary table 1. Multivariable logistic regression models evaluating factors associated with patients' first-choice follow-up preference, adjusted for demographic, clinical, and quality-of-life variables.

	**Group 1**	**Group 2**
**Variable**	**B/OR, (95% CI), p-value**	**B/OR, (95% CI), p-value**
**Age (years)**	0.105, (-0.097–0.307), 0.296	-0.082, (-0.256-0.093), 0.340
**BMI (kg/m^2)**	1.65, (1.011–2.699), **0.045**	-0.031, (-0.169-0.107), 0.640
**Education (compulsory school / university)**	-1.990, (-4.474-0.495), 0.112	-1.569, (-3.458-0.321), 0.099
**Employment (employed / unemployed)**	0.00, (0.000–0.709), **0.045**	2.420, (-1.206-6.046), 0.179
**Marital status (married or cohabiting / not married)**	0.763, (-1.503-3.030), 0.495	0.465, (-1.801-2.732), 0.673
**Comorbidities (yes/no)**	0.072, (-1.538-1.683), 0.927	-0.535, (-2.386-1.316), 0.553
**Histology (endometrioid / non-endometrioid)**	1.102, (-3.050-5.254), 0.590	-12.298, (-25.771-1.176), 0.071
**FIGO stage (I–IV)**	0.007, (-1.861-1.876), 0.994	-4.376, (-10.356-1.604), 0.143
**Adjuvant treatment (yes/no)**	-0.016, (-5.501-5.470), 0.995	-9.794, (-22.107-2.518), 0.112
**Surgery type (laparoscopic/laparotomy/vaginal)**	-0.160, (-2.099-1.778), 0.867	-0.555, (-2.323-1.212), 0.520
**Postoperative complication (yes/no)**	-1320, (-7.739-5.099), 0.676	-18.369, (-38.771-2.034), 0.113
**ASA classification (I – VI)**	-1.086, (-2.713-0.540), 0.182	0.837, (0.998-2.672), 0.353
**QLQ-Function**	-0.036, (-0.125-0.053), 0.412	-0.055, (-0.141-0.031), 0.197
**QLQ-Symptom**	0.81, (0.675–0.987), **0.036**	0.008, (-0.081-0.096), 0.855
**QLQ-Global**	0.008, (-0.069- 0.085), 0.836	0.019, (-0.047-0.085), 0.564

Footnote: Multivariable logistic regression analyses were performed separately for Group 1 and Group 2 to assess factors associated with choosing hospital visits with a doctor as the first-choice follow-up preference. Odds ratios (ORs), 95% confidence intervals (CIs), and p-values are shown. Reference categories for categorical variables are indicated in the table.

Supplementary Table 2. Association between FIGO stage and quality-of-life outcomes measured by the EORTC QLQ-C30 in Group 1 and Group 2.

**Outcome**	**Group 1**		**Group 2**	
	**B (95% CI)**	**p-value**	**B (95% CI)**	**p-value**
QLQ Function	−2.971 (−8.569 to 2.628)	0.291	−7.900 (−20.584 to 4.784)	0.215
QLQ Symptom	2.295 (−2.348 to 6.938)	0.325	4.316 (−6.629 to 15.261)	0.430
QLQ Global	−5.576 (−11.245 to 0.094)	0.054	−9.691 (−23.818 to 4.434)	0.173

Abbreviations: CI, confidence interval; FIGO, International Federation of Gynecology and Obstetrics; QLQ, Quality of Life Questionnaire. Regression coefficients (B) represent the estimated difference in quality-of-life scores associated with advanced versus early FIGO stage. Early-stage disease (FIGO I–II) was used as the reference category.
